# Uncovering hidden genetic variation in photosynthesis of field‐grown maize under ozone pollution

**DOI:** 10.1111/gcb.14794

**Published:** 2019-10-01

**Authors:** Nicole E. Choquette, Funda Ogut, Timothy M. Wertin, Christopher M. Montes, Crystal A. Sorgini, Alison M. Morse, Patrick J. Brown, Andrew D. B. Leakey, Lauren M. McIntyre, Elizabeth A. Ainsworth

**Affiliations:** ^1^ Carl R. Woese Institute for Genomic Biology University of Illinois at Urbana‐Champaign Urbana Illinois; ^2^ Department of Plant Biology University of Illinois at Urbana‐Champaign Urbana Illinois; ^3^ Department of Molecular Genetics and Microbiology University of Florida Gainesville Florida; ^4^ Genetics Institute University of Florida Gainesville Florida; ^5^ Department of Crop Sciences University of Illinois at Urbana‐Champaign Urbana Illinois; ^6^ USDA ARS Global Change and Photosynthesis Research Unit Urbana Illinois; ^7^ Present address: Department of Forest Engineering Artvin Coruh University Artvin Turkey

**Keywords:** air pollution, FACE, global climate change, heritability, maize, ozone (O_3_), photosynthesis

## Abstract

Ozone is the most damaging air pollutant to crops, currently reducing Midwest US maize production by up to 10%, yet there has been very little effort to adapt germplasm for ozone tolerance. Ozone enters plants through stomata, reacts to form reactive oxygen species in the apoplast and ultimately decreases photosynthetic C gain. In this study, 10 diverse inbred parents were crossed in a half‐diallel design to create 45 F_1_ hybrids, which were tested for ozone response in the field using free air concentration enrichment (FACE). Ozone stress increased the heritability of photosynthetic traits and altered genetic correlations among traits. Hybrids from parents Hp301 and NC338 showed greater sensitivity to ozone stress, and disrupted relationships among photosynthetic traits. The physiological responses underlying sensitivity to ozone differed in hybrids from the two parents, suggesting multiple mechanisms of response to oxidative stress. FACE technology was essential to this evaluation because genetic variation in photosynthesis under elevated ozone was not predictable based on performance at ambient ozone. These findings suggest that selection under elevated ozone is needed to identify deleterious alleles in the world's largest commodity crop.

## Graphical abstract

Ozone is the most damaging air pollutant to crops, currently reducing Midwest US maize production by up to 10%. In this study, 10 diverse inbred parents were crossed in a half‐diallel design to create 45 F1 hybrids, which were tested for ozone response in the field using Free Air Concentration Enrichment (FACE). Ozone stress increased the heritability of photosynthetic traits and altered genetic correlations among traits. FACE technology was essential to this evaluation because genetic variation in photosynthesis under elevated ozone was not predictable based on performance at ambient ozone.

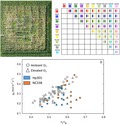

## 1 INTRODUCTION

Developing crops that can be more productive under stressful growing conditions is a high priority for agriculture today, and will be increasingly necessary if we are to avoid production losses to climate change (Challinor et al., 2014; Lesk, Rowhani, & Ramankutty, 2016; Lobell et al., 2014). Traditionally, field trials under more extreme environmental conditions than are typical for major crop growing regions have been used to test germplasm developed by breeding or biotechnology. However, some stressors—such as ozone (O_3_) pollution—are too heterogeneous and unpredictable in time or space to make this approach feasible (Ainsworth, Rogers, & Leakey, 2008). In addition, future climate change will result in growing environments with elevated [CO_2_] and temperature for which there is no present‐day analogue (Battisti & Naylor, 2009; Leakey & Lau, 2012). Controlled environment growth facilities can provide valuable information on genetic variation in crop responses to stress treatments and the mechanisms underlying genetic variation (Brosché et al., 2010; Burton, Burkey, Carter, Orf, & Cregan, 2016; Frei, Tanaka, & Wissuwa, 2008; Ueda, Siddique, & Frei, 2015), but results of such controlled environment experimentation do not always translate into improved performance under production conditions in the field (Ainsworth, Beier, et al., 2008; Araus & Cairns, 2014; McKersie, Bowley, & Jones, 1999; Passioura, 2012). Free‐air concentration enrichment (FACE) was developed to expose crops under field conditions to elevated concentrations of atmospheric pollutants over the entire growing season, with little or no perturbation to other aspects of the environment (Long, Ainsworth, Rogers, & Ort, 2004). But, most FACE experiments have tested a limited number of genotypes, at any given time (Betzelberger et al., 2010; Markelz, Strellner, & Leakey, 2011; Wang et al., 2014). Investigation of many genotypes and structured populations is needed to understand the heritability of traits in altered atmospheric environments and ultimately to identify genomic regions and genes associated with O_3_ tolerance.

Tropospheric O_3_ is a dynamic, short‐lived air pollutant that is estimated to cause annual losses of ~10% to US maize yields with crop losses of $7.2 billion (McGrath et al., 2015). However, crop yield losses to O_3_ pollution are not widely recognized by farmers. And, breeding or biotechnology for tolerance to O_3_ stress has not been a major target for seed companies (Ainsworth, 2017). Ozone is formed as a secondary pollutant from nitrogen oxides (NOx) and volatile organic compounds, and recent analyses suggest that progress towards reducing NOx in the United States has slowed considerably, thus increasing the risks of O_3_ pollution (Jiang et al., 2018). Ozone diffuses through stomatal pores on leaf surfaces and reacts to form reactive oxygen species (ROS) in the apoplast. When ROS exceed the antioxidant‐quenching capacity of the apoplast, they cause oxidative stress within cells that accelerates senescence and impairs photosynthesis, ultimately reducing plant productivity and crop yields (Ainsworth, Yendrek, Sitch, Collins, & Emberson, 2012; Kangasjärvi, Jaspers, & Kollist, 2005). Maize, like many other crop species, is sensitive to O_3_ damage, and shows accelerated loss of photosynthetic capacity with continued exposure to the air pollutant (Fiscus, Brooker, & Burkey, 2005; Yendrek, Erice, et al., 2017). Additionally, stomatal closure can be negatively impacted by O_3_ stress, leading to excessive water loss under drought stress (Wang et al., 2014; Wilkinson & Davies, 2010). Maintenance of high photosynthetic CO_2_ assimilation without excess stomatal conductance is an important phenotype for increasing O_3_ tolerance (Ainsworth, 2017; Emberson et al., 2018). Furthermore, enhancing photosynthetic CO_2_ assimilation and water use efficiency (ratio of photosynthetic CO_2_ assimilation to water loss by transpiration) are widely recognized to be key targets for crop improvement at a time when potential for further gains in harvest index and planting density may be limited (Leakey et al., 2019; Long, Marshall‐Colon, & Zhu, 2015; von Caemmerer & Furbank, 2016). Despite the importance of maize for food, fuel and animal feed, little is known about the extent or mechanisms of genetic variation in the sensitivity of maize to O_3_ by comparison to other crops such as soybean, wheat and rice (Betzelberger et al., 2012; Burton et al., 2016; Frei et al., 2008; Wang et al., 2014). This represents an important unexplored opportunity because maize is a highly tractable, model system for study of crop genetics (Buckler et al., 2009; Riedelsheimer et al., 2012; Schnable et al., 2009).

For physiological performance to be a target for improvement in breeding programmes, there must be underlying additive genetic variation in the traits of interest. The likely success of selection is reflected in the narrow sense heritability, that is, the proportion of phenotypic variation resulting from additive genetic variance (Falconer & Mackay, 1996; Flood, Harbinson, & Aarts, 2011). Previous studies of maize have estimated relatively high heritability for traits related to photosynthetic capacity (Cai et al., 2012; Crosbie, Mock, & Pearce, 1977; Lu et al., 2011; Pelleschi et al., 2006; Prado et al., 2017; Wang et al., 2013; Ziyomo & Bernardo, 2013) and indicated that variance in photosynthetic traits is mostly additive (Crosbie et al., 1977). But, the heritability of photosynthetic traits in crops is reportedly lower under stress conditions (Edwards, Ewers, McClung, Lou, & Weinig, 2012; Pelleschi et al., 2006). Prior studies on leaf‐level responses to O_3_ in fescue, potato and sweetcorn found that additive effects (GCA), not dominance effects (SCA), were significant and involved in O_3_ tolerance (De Vos, Hill, Pell, & Cole, 1982; Johnston, Haaland, & Dickens, 1983; Schraudner, Langebartels, & Sandermann, 1997). These studies fumigated crops with very high concentrations of O_3_ for hours to days, which elicited acute stress responses that are known to be fundamentally distinct from responses to season‐long, moderate O_3_ concentrations that drive yield loss in farmer's fields (Ainsworth et al., 2012; Chen, Frank, & Long, 2009; Schraudner et al., 1997). Uncertainty regarding the extent to which photosynthetic traits associated with O_3_ tolerance are heritable is compounded by the need to know if there are strong genetic correlations across environments (Falconer, 1952). In other words, if there is a substantial genotype × environment interaction acting on photosynthetic traits, then selection for crop genotypes than can tolerate elevated O_3_ pollution would not be successful under standard growing conditions. Alternatively, the absence of genotype × environment interaction would suggest that past selection for highly productive genotypes would likely have incidentally selected for tolerance to O_3_ pollution as well. In addition, genetic correlations among traits are useful in assessing how many independent traits need to be evaluated for a successful selection index to be developed.

Proof‐of‐concept is needed to demonstrate the use of FACE experimentation to estimate the heritability of photosynthetic traits and the degree to which elevated O_3_ affects heritability in a farm‐field setting (Frei, 2015). Therefore, in this study, we used a half‐diallel mating design to test for (a) the effects of elevated O_3_ on photosynthetic traits in maize; (b) the heritability and genetic correlations among photosynthetic traits in maize; and (c) the identification of particularly susceptible parental lines.

## 2 MATERIALS AND METHODS

### 2.1 Field site and experimental treatments

**Figure 1:**
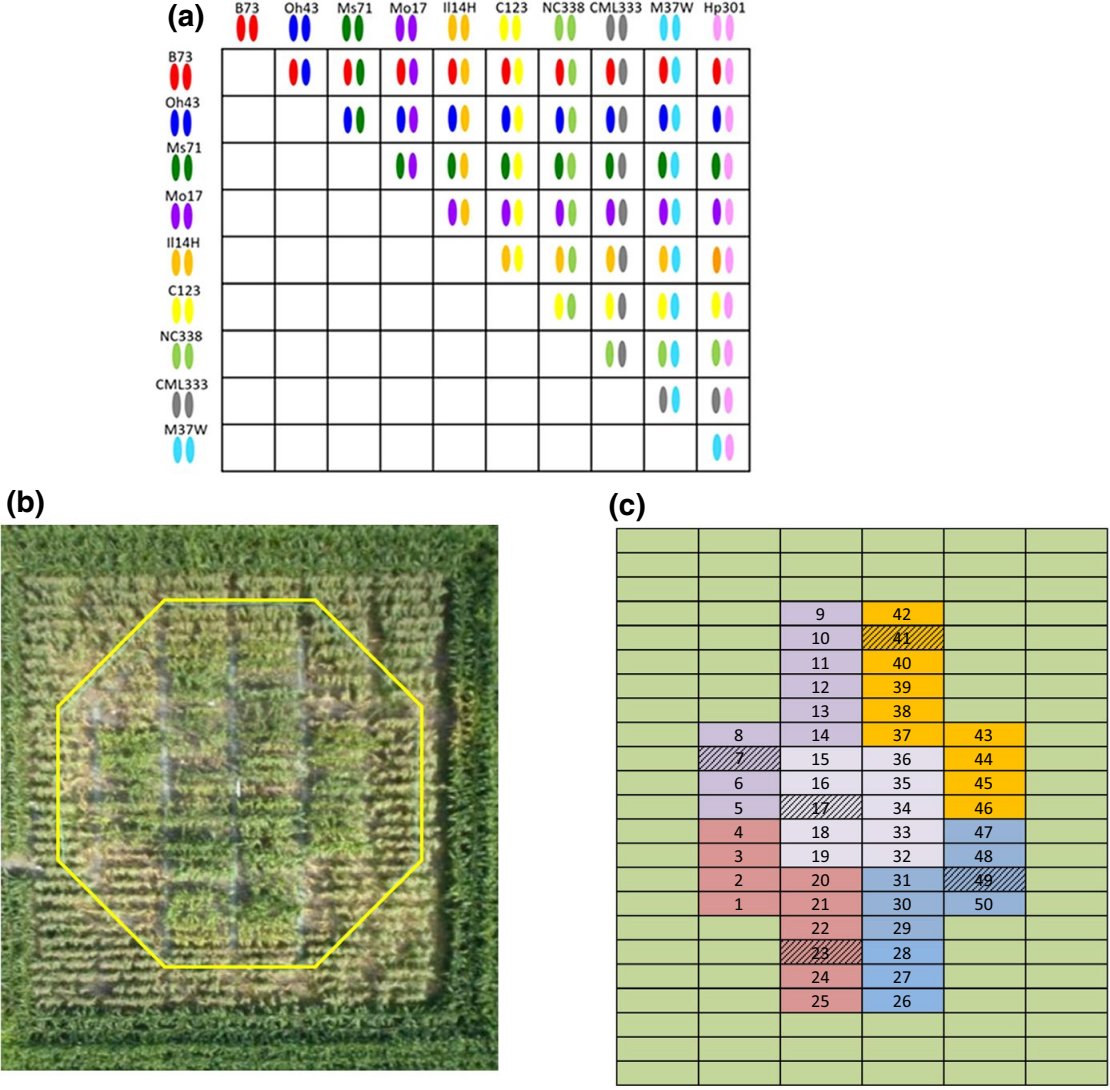
(a) Half‐diallel design of 45 F_1_ hybrids tested for response to elevated O_3_. (b) Aerial image of an elevated O_3_ ring with yellow indicating the pipes from which air enriched with O_3_ is released into the wind. (c) The spatial layout of a ring with different colour shading indicating the five sub‐blocks within the ring. Nine genotypes were assigned to a ‘set’ and a common genotype (B73 × Mo17) was replicated in each set as a spatial check (hashed boxes). Sets were randomly assigned to different sub‐blocks in each of the four replicate ambient and elevated O_3_ rings

In 2016 and 2017, a maize half‐diallel panel of 45 crosses among 10 maize inbred lines (Figure 1) was planted at the FACE field site located on the experimental farms of the University of Illinois at Urbana‐Champaign (40°02′N, 88°14′W; http://www.igb.illinois.edu/soyface/). Seeds were planted on May 24, 2016 and May 17, 2017. Experimental rows were 3.35 m long with a planting density of 8 plants/m and row spacing of 0.76 m. Maize was grown in rotation with soybean, fertilized with N (200 kg/ha) and irrigated as needed using drip irrigation. Maize was exposed to ambient or elevated O_3_ in 20 m dia. octagonal rings (*n* = 4 paired ambient and elevated O_3_ rings). Each ring was divided into five sub‐blocks of 10 rows each (Figure 1). The 45 genotypes were randomized into five entry sets of nine genotypes and entry‐sets were randomly assigned to different sub‐blocks in each of the four pairs of rings (one ambient, one elevated). Within each sub‐block, B73 × Mo17 was planted as a check genotype.

The O_3_ treatment was applied at a target set point of 100 nl/L from 10:00 to 18:00 throughout the growing season when it was not raining, when leaves were dry and when wind speed was greater than 0.5 m/s (Yendrek, Erice, et al., 2017; Yendrek, Tomaz, et al., 2017). Based on 1 min average O_3_ concentration collected in each ring throughout the season, the fumigation was within 10% of the 100 nl/L target for 62% of the time and within 20% of the target concentration for 87% of the time in 2016. In 2017, fumigation was within 10% of the target for 59% of the time and within 20% of the target for 84% of the time. Other meteorological conditions were measured with an on‐site weather station (Figure S1).

### 2.2 Measurements of photosynthetic traits

On July 5–9, 2016 and June 28–July 1, 2017 leaf gas exchange was measured. In 2016, the eighth leaf was measured (approximately the third or fourth fully expanded leaf), while in 2017 the third fully collared leaf was measured. Prior to dawn, leaves from two plants per genotype from one ambient and one elevated O_3_ plot were labelled and cut, providing ~208 leaves for measurements per day. Leaves were immediately placed in a bucket with the cut surface submerged in water, transported to a laboratory, recut under water and placed in 50 ml tubes filled with water. Leaves were stored in dim light (<100 µmol m^−2^ s^−1^). Approximately 1 hr before the measurement of leaf gas exchange, leaves were placed in a growth chamber with the following conditions: 1,800 photosynthetic photon flux density (PPFD, µmol m^−2^ s^−1^) at the leaf surface, 18°C air temperature (25°C leaf temperature) and relative humidity of ~90%. After approximately 1 hr of acclimation, leaf gas exchange was measured with a set of portable gas exchange systems (LI‐6400; LI‐COR Biosciences, Lincoln, NE, USA) with 2 × 3 red/blue LED cuvettes (LI‐6400‐02B; LI‐COR Biosciences). Light‐saturated gas exchange was measured at 1,800 PPFD (µmol m^−2^ s^−1^), 25°C (leaf temperature) and 60% relative humidity.

Gas exchange systems were run simultaneously, and leaf mean flux of net CO_2_ assimilation (*A*), stomatal conductance (*g*_s_) and the ratio of the leaf intercellular concentration of CO_2_ to atmospheric CO_2_ (*c*_i_:*c*_a_) were measured or calculated every 4 s for 4 min (Figure S2). Analysis was done on the average values of *A*, *g*_s_ and *c*_i_:*c*_a_ over the last minute of measurement. Instantaneous water use efficiency (iWUE = *A*/*g*_s_) was calculated from *A* and *g*_s_. Following gas exchange measurements, leaf reflectance was measured from the adaxial surface of the leaf using a full‐range spectroradiometer (ASD FieldSpec 4 Standard Res, Analytical Spectral Devices) following a standard protocol (Yendrek, Erice, et al., 2017; Yendrek, Tomaz, et al., 2017). Six reflectance measurements were collected and a splice correction was applied to the spectra to ensure continuous data across detectors (Serbin, Singh, McNeil, Kingdon, & Townsend, 2014). Those six spectra from each leaf were then averaged and data were interpolated to provide 1 nm bandwidths. Further quality control was applied using the FieldSpectra package in R (Serbein et al., 2014). Partial least squares regression models were then applied to the spectra to estimate the CO_2_‐saturated rate of *A* (*V*_maxm_) and leaf chlorophyll content (Chl_m_; Yendrek, Erice, et al., 2017; Yendrek, Tomaz, et al., 2017).

### 2.3 Data quality control

Measurements of gas exchange over time were examined for data quality and conformation to basic assumptions. If mean *g*_s_ or *c*_i_ for the last minute of the measurement period were negative, then data were not used (Figure S2). Individual leaf measurements for the same genotype and treatment were evaluated for outliers and influential observations. If an individual measurement strongly influenced the stability of the data, the observation was not used for further statistical analysis.

### 2.4 Statistical analysis

B73 × Mo17 was planted within each sub‐block of the ring (Figure 1), and used as a covariate in the model (see Supplemental Text for additional statistical tests). As the majority of traits showed evidence for interactions between additive genetic variance and an interaction between additive genetic variance and O_3_ treatment, the ambient and elevated O_3_ environments were modelled separately to estimate heritability:

$$y_{\mathrm{ijkm}}=\mu_{i}+C_{\mathrm{ijk}}+G_{m}+\varepsilon_{\mathrm{ijkm}}.$$

$y_{\mathrm{ijkm}}$ is the observed trait value for the *i*th treatment (ambient or elevated O_3_) in the *k*th sub‐block of the *j*th ring‐pair, belonging to the cross from the *m*th mother where *µ_i_* is the mean for the *i*th treatment. $C_{\mathrm{ijk}}$ is the trait value of the B73 × Mo17 check plot in the *i*th treatment, *j*th ring‐pair and *k*th sub‐block. $G_{m}$ is the random GCA effect (parameterized as a set of indicator variables for the maternal parent). The variance component for GCA was estimated using a Toeplitz(1) variance structure (Wayne et al., 2007).

Narrow sense heritability and standard error (*SE*) were estimated for each trait in ambient and elevated O_3_ conditions separately as:

$$h_{n}^{2}=\frac{2\;*\;\sigma_{\text{gca}}^{2}}{2\;*\;\sigma_{\text{gca}}^{2}+\sigma_{e}^{2}},$$

where $\sigma_{\text{gca}}^{2}$ estimated variance of GCA effect and $\sigma_{e}^{2}$ is residual variance. The *SE* of heritability estimates was obtained with the Delta method (Wayne et al., 2007).

Genetic correlations between environments for the same trait (*r*_g_amb_oz_) were calculated using a MANOVA based on the above model where the two dependent variables were the genotypic mean values in each of the two environments:

$$Y_{i}=\left[\begin{array}{c} w_{\text{amb}}\\ w_{\text{oz}} \end{array}\right],\;\varepsilon_{i}=\left[\begin{array}{c} \varepsilon_{\text{amb}}\\ \varepsilon_{\text{oz}} \end{array}\right]$$

$$Z_{i}=∼\left(0,\left[\begin{array}{cc} \sigma_{{\text{gca}}_{\text{amb}}}^{2} & \sigma_{{\text{gca}}_{\text{amb}\_\text{oz}}}\\ \sigma_{{\text{gca}}_{\text{amb}\_\text{oz}}} & \sigma_{\sigma_{{\text{gca}}_{\text{oz}}}}^{2} \end{array}\right]\right).$$

The genetic correlation between environments for the same trait was calculated from estimates in the above model as:

$$r_{g_{\text{amb\textbackslash{}\_oz}}}=\frac{\sigma_{{\text{gca}}_{\text{amb\textbackslash{}\_oz}}}}{\sqrt{\sigma_{{\text{gca}}_{\text{amb}}}^{2}\;*\;\sigma_{{\text{gca}}_{\text{oz}}}^{2}}},$$

where $\sigma_{{\text{gca}}_{\text{amb\textbackslash{}\_oz}}}$ is the genetic covariance between environments for a given trait and $\sigma_{{\text{gca}}_{\text{amb}}}^{2}$ and $\sigma_{{\text{gca}}_{\text{oz}}}^{2}$ are the additive genetic variances for the same trait in ambient and elevated O_3_ respectively.

Genetic correlations between traits within an O_3_ treatment (*r_g_*__trait1_trait2_) were calculated based on the above model using a MANOVA approach where a pair of traits was considered within the same environment:

$$Y_{i}=\left[\begin{array}{c} w_{\text{trait}1_{\text{oz}}}\\ w_{\text{trait}2_{\text{oz}}} \end{array}\right],\;\varepsilon_{i}=\left[\begin{array}{c} \varepsilon_{\text{trait}1_{\text{oz}}}\\ \varepsilon_{\text{trait}2_{\text{oz}}} \end{array}\right]$$

$$\gamma_{i}=\left[\begin{array}{c} \gamma_{\text{trait}1_{\text{oz}}}\\ \gamma_{\text{trait}2_{\text{oz}}} \end{array}\right]=\;∼\left(0,\left[\begin{array}{cc} \sigma_{{\text{gca}}_{\text{trait}1\_\text{oz}}}^{2} & \sigma_{{\text{gca}}_{\text{trait}1\_\text{oz}\_\text{trait}2\_\text{oz}}}\\ \sigma_{{\text{gca}}_{\text{trait}1\_\text{oz}\_\text{trait}2\_\text{oz}}} & \sigma_{{\text{gca}}_{\text{trait}2\_\text{oz}}}^{2} \end{array}\right]\right).$$

Genetic correlations between traits for each environment were calculated as:

$$r_{g_{\text{trait}1\_\text{trait}2_{\text{oz}}}}=\frac{\sigma_{{\text{gca}}_{\text{trait}1\_\text{oz}\_\text{trait}2\_\text{oz}}}}{\sqrt{\sigma_{{\text{gca}}_{\text{trait}1\_\text{oz}}}^{2}\;*\;\sigma_{{\text{gca}}_{\text{trait}2\_\text{oz}}}^{2}}},$$

where $\sigma_{{\text{gca}}_{\text{trait}1\_\text{oz}\_\text{trait}2\_\text{oz}}}$ is the additive genetic covariance between traits for the same environment, and $\sigma_{{\text{gca}}_{\text{trait}1\_\text{oz}}}^{2}$ and $\sigma_{{\text{gca}}_{\text{trait}2\_\text{oz}}}^{2}$ are the additive genetic variances for trait1 and trait2.

Phenotypic correlations within an O_3_ treatment (*r*_P_trait1_trait2_) were calculated as:

$$r_{P_{\text{trait}1\_\text{trait}2_{\text{oz}}}}=\frac{\sigma_{P_{\text{trait}1\_\text{oz}\_\text{trait}2\_\text{oz}}}}{\sqrt{\sigma_{P_{\text{trait}1\_\text{oz}}}^{2}\;*\;\sigma_{P_{\text{trait}2\_\text{oz}}}^{2}}},$$

where $\sigma_{P_{\text{trait}1\_\text{oz}\_\text{trait}2\_\text{oz}}}=\sigma_{{\text{gca}}_{\text{trait}1\_\text{oz}\_\text{trait}2\_\text{oz}}}+\sigma_{e_{\text{trait}1\_\text{oz}\_\text{trait}2\_\text{oz}}}$. All calculations were performed using code adapted from Holland (2006).

Visual examination of the results led to the hypothesis that NC338 and HP301 were responsible for the shift in genetic correlations. Thus, the correlation analyses described above were repeated for the set of F_1_ hybrids that did not include hybrids NC338 or Hp301 as either parent.

## 3 RESULTS

### 3.1 Gas exchange response to elevated O_3_

**Figure 2:**
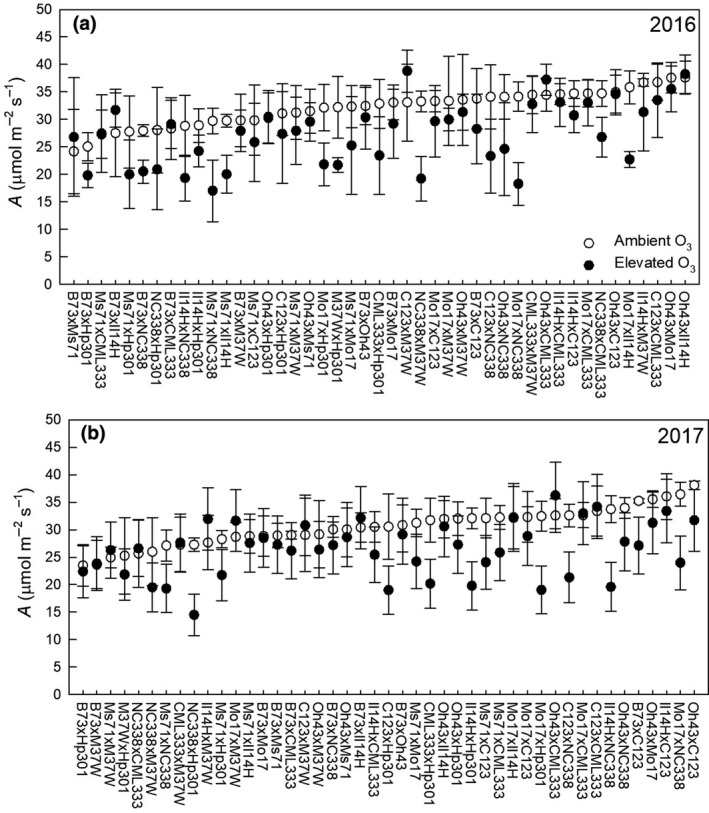
Measurements of net carbon assimilation (*A*) measured in the F_1_ maize hybrids. Measurements were made during the summer of 2016 (a) and 2017 (b) on hybrids grown in ambient O_3_ (white) and elevated O_3_ (black). Data are ordered based on *A* measured at ambient O_3_. Error bars represent 1 *SD*

Photosynthetic traits of 45 F_1_ hybrids comprising a half‐diallel population were measured in ambient and elevated O_3_ in 2016 and 2017. Measurements were taken on mature leaves approximately midway through vegetative development of the crop in both years. Rainfall was lower in 2017, leading to greater drought stress than in 2016 (Figure S2), and lower *A* and *g*_s_ (Figure 2; Figure S3). In both years, averaging across hybrids, elevated O_3_ reduced *A*, by ~14% (Figure 2), and *g*_s_ by 6% in 2016 and by 14% in 2017 (Figure S3).

### 3.2 Narrow sense heritability increases with O_3_ environment

Analysis of the half‐diallel maize population grown showed that additive genetic variance explained the variation in photosynthetic and reflectance traits (Table S1). There was little evidence for dominance variance (Table S1; comparison of models 2 and 3; Table S2). In addition, there was strong evidence for interactions between genetic variation and environment (G × E; Table S1; comparison of models 2 and 3), indicating that the genetic architecture of most traits was different in the two environments. Therefore, heritability was calculated separately in ambient and elevated O_3_. Narrow sense heritability ($h_{n}^{2}$) estimates of photosynthetic traits differed between ambient and elevated O_3_ in 2016 and 2017 (Table 1). Interestingly, $h_{n}^{2}$ in elevated O_3_ was greater than in ambient O_3_ for many photosynthetic traits (Table 1), indicating the presence of additional additive genetic variation in elevated O_3_. Estimated $h_{n}^{2}$ of *A* was greater than $h_{n}^{2}$ of other photosynthetic traits (Table 1), and was of similar magnitude to previous estimates in maize (Crosbie et al., 1977; Pelleschi et al., 2006).

**Table 1:** Narrow sense heritability ($h_{n}^{2}$) for photosynthetic traits measured in 2016 and 2017 in ambient and elevated [O_3_]

	2016		2017	
	Ambient O_3_	Elevated O_3_	Ambient O_3_	Elevated O_3_
*A*	0.222	0.678	0.315	0.499
*g*_s_	0.178	0.380	0.278	0.202
iWUE	0.127	0.182	0.212	0.203
*c*_i_:*c*_a_	0.103	0.182	0.197	0.237
*V*_maxm_	0.125	0.236	0.197	0.258
Chl_m_	0.424	0.283	0.171	0.276

### 3.3 Elevated O_3_ alters genetic and phenotypic correlations among photosynthetic traits

**Figure 3:**
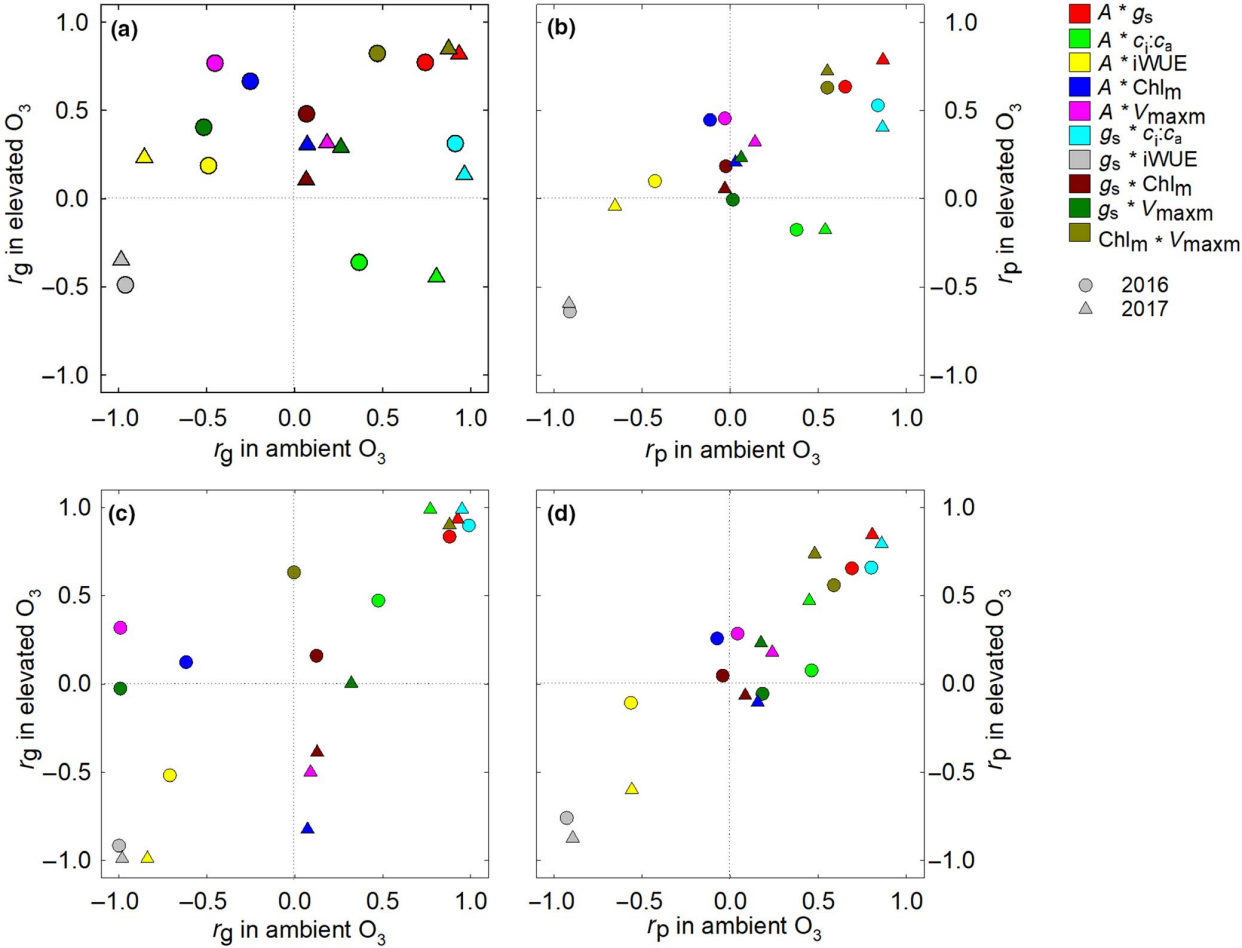
Genetic and phenotypic correlations among photosynthetic traits. Genetic correlations (a, c) and phenotypic correlations (b, d) among photosynthetic traits in ambient and elevated O_3_ calculated in 2016 and 2017. Top panels include all hybrids (a, b). Bottom panels show correlations after hybrids with parents Hp301 or NC338 were removed from the analysis (b, c)

Genetic correlations (*R*_g_) are important to define the shared genetic components between traits and phenotypic correlations (*R*_p_) measure the consistency of performance between traits. Both *R*_g_ and *R*_p_ between *A* and *g*_s_ were very strong regardless of the environment or year (Figure 3a,b, red symbols). This is unsurprising because *g*_s_ is a principle determinant of CO_2_ entry into leaves, and *A* measures CO_2_ fixation. *R*_g_ between *g*_s_ and iWUE were strongly negative in both 2016 and 2017, indicating that selection for high *g*_s_ in maize would result in low iWUE (Figure 3, grey symbols). There was a positive *R*_g_ between traits estimated from leaf reflectance spectra, Chl_m_ and *V*_maxm_ (Figure 3a, olive symbols) in both ambient and elevated O_3_, but *R*_g_ between *A* and *V*_maxm_ or Chl_m_ was not strong or consistent (Figure 3a, pink symbols). It was notable that *R*_g_ between *A* and *c*_i_:*c*_a_ (Figure 3a, green symbols) and *g*_s_ and *c*_i_:*c*_a_ (Figure 3a, cyan symbols) differed in ambient and elevated O_3_. *R*_g_ between *g*_s_ and *c*_i_:*c*_a_ was strong and positive in ambient O_3_, but not in elevated O_3_ (Figure 3a, cyan symbols), and *R*_g_ between *A* and *c*_i_:*c*_a_ was positive in ambient O_3_ and negative in elevated O_3_ (Figure 3a, green symbols). These same general trends were also observed for *R*_p_ (Figure 3b).

### 3.4 Identification of O_3_‐sensitive maize lines

**Figure 4:**
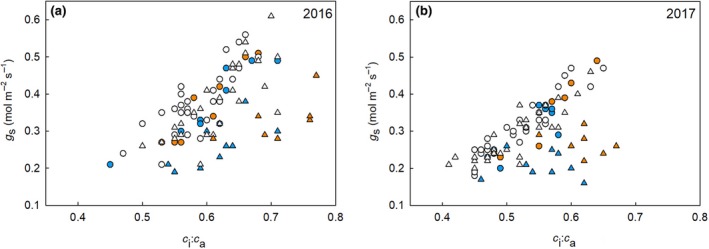
The relationship between stomatal conductance (*g*_s_) and the ratio of the leaf intercellular concentration of CO_2_ to atmospheric CO_2_ (*c*_i_:*c*_a_) in maize hybrids grown at ambient (circles) and elevated O_3_ (triangles) in 2016 (a) and 2017 (b). Blue symbols represent hybrids with Hp301 as a parent and orange symbols hybrids with NC338 as a parent

**Figure 5:**
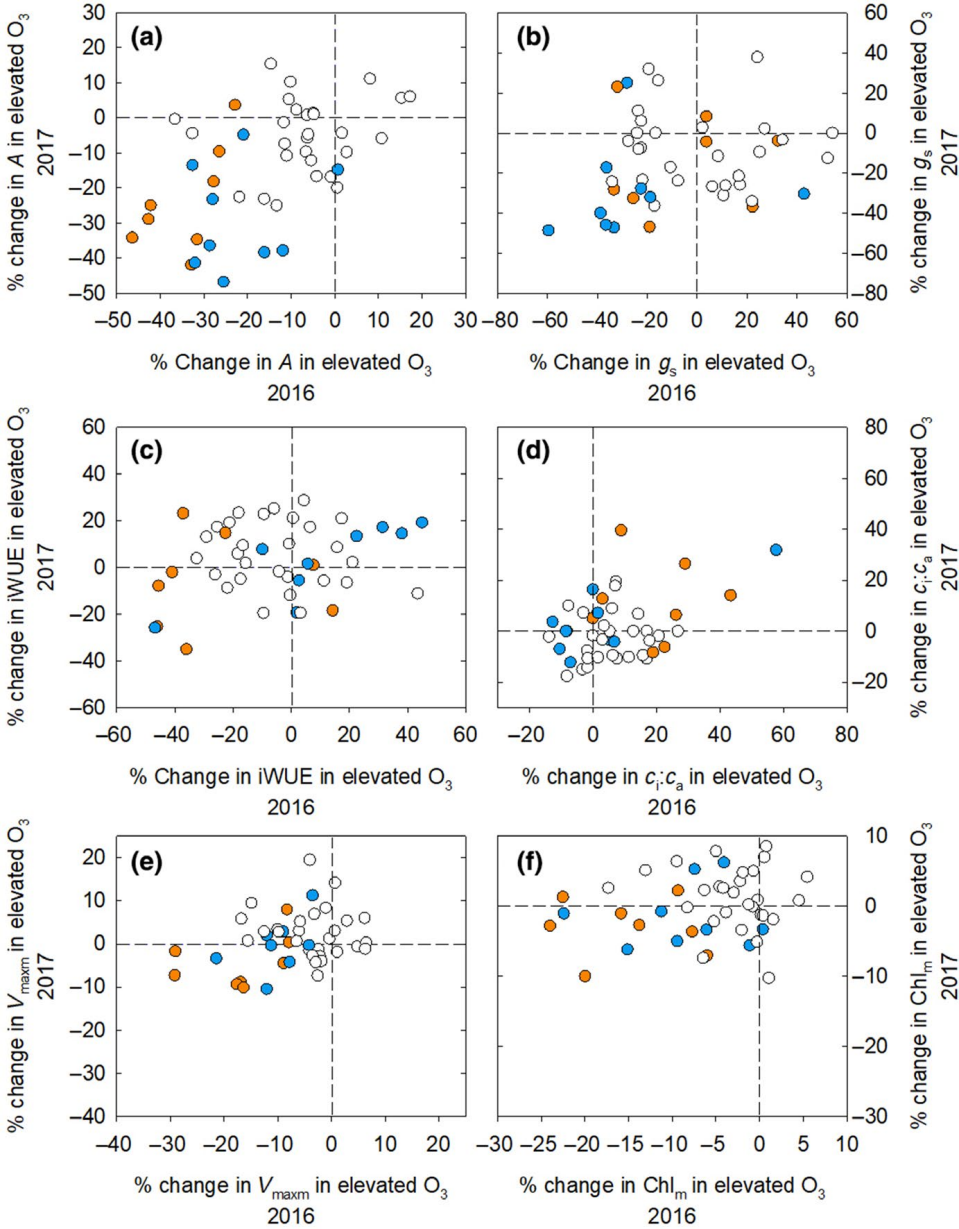
Per cent change of photosynthetic traits at elevated O_3_. Blue circles represent hybrids with Hp301 as the male or female parent and orange circles are hybrids with NC338 as the male or female parent

The correlation between *g*_s_ and *c*_i_:*c*_a_ was further explored based on the differences in *R*_g_ between traits in ambient and elevated O_3_. Across the diverse F_1_ hybrids, there was a strong, linear relationship between *g*_s_ and *c*_i_:*c*_a_ consistent among all lines in ambient O_3_ (Figure 4, circles). However, growth at elevated O_3_ altered the relationship between *g*_s_ and *c*_i_:*c*_a_ in F_1_ crosses with parents NC338 (Figure 4, orange triangles) and Hp301 (Figure 4, blue triangles) in both 2016 and 2017. Notably, alleles from Hp301 and NC338 appear to confer sensitivity to elevated O_3_ in different ways. Lower *A* at elevated O_3_ in hybrids with Hp301 as a parent was linked to lower *g*_s_ at elevated O_3_ without a change in *c*_i_:*c*_a_ (Figure 4). Meanwhile, lower *A* at elevated O_3_ in hybrid with NC338 as a parent was linked to greater *c*_i_:*c*_a_ without a change in *g*_s_ (Figure 4). F_1_ hybrids with these two parents also showed the greatest per cent decrease in *A* and *g*_s_ at elevated O_3_ in 2016 and 2017 (Figure 5a,b). When *R*_g_ and *R*_p_ were estimated without hybrids containing NC338 or Hp301 (Figure 3c,d), then estimates were similar in both ambient and elevated O_3_. This further indicates that F_1_ crosses with Hp301 and NC338 are sensitive to O_3_ and drive the differences in *R*_g_ and *R*_p_ observed between *A* and *c*_i_:*c*_a_ and *g*_s_ and *c*_i_:*c*_a_.

## 4 DISCUSSION

This 2‐year study enabled investigation of genetic variation in photosynthetic traits as well as the consistency of the response of photosynthetic traits to elevated O_3_ across growing seasons. Photosynthesis has been suggested as one of the most important processes to improve in order to combat stagnating crop yields and improve future food security (Long et al., 2015). Transgenic approaches to improve C4 photosynthesis have been suggested (von Caemmerer & Furbank, 2016), but exploiting standing genetic variation in photosynthetic traits is also a path to crop improvement that does not involve transgenic technology (Cañas et al., 2017). Here, we showed that maize hybrids have significant additive genetic variation in *A* in both ambient and elevated O_3_ (Figure 2), and the per cent change in *A* at elevated O_3_ was consistent in both years (Figure 5a). By using a half‐diallel mating design comprised of diverse maize lines, we estimated genetic (*R*_g_) and phenotypic (*R*_p_) correlations in both environments and demonstrated that growth at elevated O_3_ alters the *R*_g_ and *R*_p_ between photosynthetic traits. Growth at elevated O_3_ changes the relationship between *g*_s_ and *c*_i_:*c*_a_, primarily driven by genotypes with Hp301 and NC338. Furthermore, the mechanisms of sensitivity to elevated O_3_ appear to differ between genotypes Hp301 and NC338. Overall, we have demonstrated the utility of FACE experiments for screening structured populations to estimate heritability of phenotypic traits and how those change with O_3_ pollution. This capacity and information is critical for any future efforts to breed for O_3_ tolerance.

Previous studies of O_3_‐induced leaf damage in fescue, tobacco, maize, potato and *Plantago major* also showed little evidence of dominance variance (Aycock, 1972; Cameron, 1975; Campbell, Devine, & Howelp, 1977; De Vos et al., 1982; Huang, Aycock, & Mulchi, 1975; Johnston et al., 1983; Whitfield, Davison, & Ashenden, 1997), which is consistent with our findings. Lack of dominance variance indicates that the narrow sense heritability ($h_{n}^{2}$) of net carbon assimilation (*A*) in elevated O_3_ is mainly additive and can be attributed to additive genetic factors inherited from the parents. The observed increase in $h_{n}^{2}$ of *A* under O_3_ stress is notable. A prior study investigating O_3_ sensitivity in Black Cherry (*Prunus serotine* Ehrh.) also found that heritability of the foliar injury increased with exposure to greater treatment concentrations of elevated O_3_ (Lee, Steiner, Zhang, & Skelly, 2002). In contrast, studies that investigated other abiotic stresses such as drought stress often reported that $h_{n}^{2}$ was lower under stress conditions. For example, drought stress decreased $h_{n}^{2}$ of *A* from 0.61 to 0.33 in *Brassica rapa* (Edwards et al., 2012) and from 0.45 to 0.37 in maize (Pelleschi et al., 2006). Greater $h_{n}^{2}$ in elevated O_3_ as revealed in this study suggests that there is potential for improvement. Furthermore, selection for *A* under standard growing conditions of the Midwest in the United States would fail to select against deleterious alleles that confer sensitivity to elevated O_3_ (Gibson & Dworkin, 2004).

Genetic correlations (*R*_g_) among traits imply that the same genes are acting on multiple traits. If the correlation is high enough, it is possible that selection can be performed on one trait, with the second trait also improving due to shared loci between the traits. We tested *R*_g_ among photosynthetic traits measured with gas exchange and traits estimated from leaf hyperspectral reflectance (Yendrek, Erice, et al., 2017; Yendrek, Tomaz, et al., 2017), which has been suggested as a promising high‐throughput approach for phenotyping (Araus & Cairns, 2014; Furbank & Tester, 2011). Although *R*_g_ between *V*_maxm_ and Chl_m_ estimated from hyperspectral reflectance was strong and positive, *R*_g_ between gas exchange traits and remotely sensed traits was not strong in our experiments (Figure 3a). This could be because chlorophyll content and maximum photosynthetic capacity were not the primary limitations to gas exchange at the times of our measurement. In 2017, reflectance was measured in the field on intact plants, which may also lead to lower correlations. We did observe strong, positive correlations between *A* and *g*_s_, which has been reported previously for other species under variable environmental conditions (Manzaneda, Rey, Anderson, Raskin, & Mitchell, 2016; Pelleschi et al., 2006). We also identified a fundamental shift in *R*_g_ between *g*_s_ and *c*_i_:*c*_a_ in ambient and elevated O_3_ (Figure 3a). *c*_i_:*c*_a_ provides information on the balance between resistance for CO_2_ diffusion into the leaf and the biochemical capacity for CO_2_ fixation in the mesophyll. Altered *R*_g_ in elevated O_3_ suggests the involvement of additional genetic factors in controlling the phenotype under O_3_ stress.

Further analysis of the relationship between *g*_s_ and *c*_i_:*c*_a_ identified that hybrids with NC338 and Hp301 were more sensitive to O_3_ stress (Figure 4). When *R*_g_ and *R*_p_ were calculated without hybrids containing Hp301 and NC338, the correlations become similar in ambient and elevated O_3_ (Figure 3c,d), suggesting that alleles from Hp301 and NC338 were responsible for the change in genetic architecture of photosynthetic traits in elevated O_3_. These alleles from Hp301 and NC338 demonstrate different mechanisms of sensitivity to elevated O_3_, with Hp301 crosses linked to lower *g*_s_ at elevated O_3_ with no change in *c*_i_:*c*_a_ and NC338 hybrids showing greater *c*_i_:*c*_a_ without a change in *g*_s_ (Figure 5). This implies that stomatal function was disrupted in Hp301 lines, which is consistent with the prior evidence that O_3_ stress alters stomatal signalling and closure (Wilkinson & Davies, 2010). Meanwhile, our findings suggest that alterations in mesophyll conductance (*g*_m_) or perhaps decreases in photosynthetic capacity drove sensitivity to elevated O_3_ in hybrids with NC338 as a parent. This is consistent with greater average reductions in *V*_maxm_ and Chl_m_ in hybrids containing NC338 as a parent (Figure 5). A study of Siebold's beech (*Fagus crenata*) seedlings found a significant increase in *c*_i_ and a substantial decrease in *g*_m_ with increasing O_3_ stress, suggesting that lower *g*_m_ hindered CO_2_ transfer to the site of carbon fixation (Watanabe et al., 2018). Similarly, a study on snap peas found the genotype sensitive to elevated O_3_ had a significant decrease in *g*_m_ (Flowers, Fiscus, Burkey, Booker, & Dubios, 2007). Photosynthetic processes may be sensitive to O_3_ (Fiscus et al., 2005) and reductions in PEPc activity and Rubisco activity were reported at elevated O_3_ in young maize leaves (Leitao, Bethenod, & Biolley, 2007). Such reductions in either *g*_m_ or photosynthetic enzyme activity if decoupled from *g*_s_ could increase *c*_a_, as observed in the sensitive NC338 hybrids here.

Global mean O_3_ pollution increased by 8.9% from 1990 to 2013 (Brauer et al., 2016) causing a significant, negative impact on crop production in North America and Europe worldwide (Mills et al., 2018). Identifying genetic variation in photosynthetic traits and tolerant genotypes to elevated O_3_ is a crucial step towards breeding for O_3_ tolerance (Ainsworth, 2017). For breeding to be straightforward, genetic variation should be directly available to selection, and the genetic contribution to phenotypic variation should be additive. This first investigation of a half‐diallel population of maize exposed to elevated O_3_ in the field showed not only that additive genetic variation is a large component of phenotypic variation in photosynthetic traits, but also that O_3_ stress increased the heritability of photosynthetic traits, indicating additional loci responding to O_3_ stress. In addition, the genetic correlation among photosynthetic traits changed between environments. The F_1_ hybrids from parents Hp301 and NC338 were particularly sensitive to O_3_ and showed disrupted relationships between *A* or *g*_s_ and *c*_i_:*c*_a_. The physiological mechanism underlying this sensitivity was distinct between the two parent lines, suggesting that multiple sources of sensitivity to oxidative stress from air pollution might need to be selected against to optimize maize production. This implies that past selection of maize under ambient O_3_ did not select for alleles that confer tolerance to elevated O_3_ pollution. Further selection under elevated O_3_ concentrations might purge deleterious alleles in the world's most important commodity crop. More broadly, this work demonstrates the capability of FACE technology to be used for field experimentation on diverse populations of major commodity crops to address needs for adaptation to pollution and climatic change that cannot be easily accomplished by other existing approaches.
