# Supplementary material for: Uncovering hidden genetic variation in photosynthesis of field‐grown maize under ozone pollution
Source: Glob Chang Biol. 2019 Oct 1;25(12):4327–38. doi: 10.1111/gcb.14794 (PMC6899704; doi:10.1111/gcb.14794)
Supplement: Supplementary file 1 [file GCB-25-4327-s001.docx]

**Supplemental Text**

**Statistical Analysis**

Entry sets did not contribute significantly to variance in any trait, and were not considered in statistical models. To account for spatial variation within the field and variation in O_3_ fumigation within the ring, different models were tested. Models with the effect of ring-pair and sub-block were fit and compared to models that used the B73xMo17 hybrid check as a covariate (see Figure 1 for experimental design). The models performed similarly and no impact on inference was detected (Supplemental Figure 4). Therefore, we present the models using the B73xMo17 check as a covariate as these models most accurately describe the augmented field design.

We are describing our models using the linear models description proposed by Searle (1971) where the model parameters are not explicitly shown and the value in the subscripts reflect the values in the data matrix. In order to determine if there was evidence for dominance variance, two models were fit: a model containing dominance effects (SCA) and a model with only additive effects (GCA). A model (*Model 1*) with dominance effects was fit as follows:

$\boldsymbol{y}_{\boldsymbol{ijkmn}}\boldsymbol{=u+}\boldsymbol{T}_{\boldsymbol{i}}\boldsymbol{+}\boldsymbol{C}_{\boldsymbol{ijk}}\boldsymbol{+}\boldsymbol{G}_{\boldsymbol{m}}\boldsymbol{+}\boldsymbol{S}_{\boldsymbol{n}\left( \boldsymbol{m} \right)}\boldsymbol{+}\boldsymbol{\epsilon}_{\boldsymbol{ijkmn}}$ **(1)**

in which $\boldsymbol{y}_{\boldsymbol{ijkmn}}$, is the observed trait value for the *i^th^* treatment (ozone, ambient) in the *k^th^* sub-block of the *j^th^* ring-pair, belonging to the cross from the *m^th^* mother and *n^th^* father. $\boldsymbol{u}$ is the overall mean effect, $\boldsymbol{C}_{\boldsymbol{ijk}}$, is the trait value of the B73xMo17 check plot in the *i^th^* treatment, *j^th^* ring-pair and *k^th^* sub-block. $\boldsymbol{G}_{\boldsymbol{m}}$ is the random GCA effect (parameterized as a set of indicator variables for the maternal parent), $\boldsymbol{S}_{\boldsymbol{n(m)}}$, is random SCA effect (parameterized as a categorical variable indicating the cross 1 to 45). The variance component for GCA was estimated using a Toeplitz(1) variance structure. A model with just additive effects was fit (*Model 2*) as follows:

$\boldsymbol{y}_{\boldsymbol{ijkm}}\boldsymbol{=u+}\boldsymbol{T}_{\boldsymbol{i}}\boldsymbol{+}\boldsymbol{C}_{\boldsymbol{ijk}}\boldsymbol{+}\boldsymbol{G}_{\boldsymbol{m}}\boldsymbol{+}\boldsymbol{\epsilon}_{\boldsymbol{ijkm}}$ **(2)**

Bayesian information criterion (BIC) values were used in the evaluation of model fit (Schwarz 1978; Coffman et al. 2005). The two models were compared using a likelihood ratio test (LRT) to determine whether there was evidence for SCA, and a Wald Test of the null hypothesis that the random effect of SCA was zero (Supplemental Table 1). To examine whether there was evidence for an interaction between GCA and the environment, we compared *Model 2* to *Model 3:*

$\boldsymbol{y}_{\boldsymbol{ijkm}}\boldsymbol{=u+}\boldsymbol{T}_{\boldsymbol{i}}\boldsymbol{+}\boldsymbol{C}_{\boldsymbol{ijk}}\boldsymbol{+}\boldsymbol{G}_{\boldsymbol{m}}\boldsymbol{+}{\boldsymbol{T}_{\boldsymbol{i}}\boldsymbol{*G}}_{\boldsymbol{m}}\boldsymbol{+}\boldsymbol{\epsilon}_{\boldsymbol{ijkm}}$ **(3)**

using a LRT. We estimated the *p*-value using a Wald test of the null hypotheses that the variance component is zero. We attempted to fit a full model (*Model 4*) as follows:

$\boldsymbol{y}_{\boldsymbol{ijkmn}}\boldsymbol{=u+}\boldsymbol{T}_{\boldsymbol{i}}\boldsymbol{+}\boldsymbol{C}_{\boldsymbol{ijk}}\boldsymbol{+}\boldsymbol{G}_{\boldsymbol{m}}\boldsymbol{+}{\boldsymbol{T}_{\boldsymbol{i}}\boldsymbol{*G}}_{\boldsymbol{m}}\boldsymbol{+}\boldsymbol{S}_{\boldsymbol{n(m)}}\boldsymbol{+}\boldsymbol{T}_{\boldsymbol{i}}\boldsymbol{*}\boldsymbol{S}_{\boldsymbol{n(m)}}\boldsymbol{+}\boldsymbol{\epsilon}_{\boldsymbol{ijkmn}}$ **(4)**

This model failed to converge for the majority of traits and was not considered further. In all comparisons of models, the *p* values for the LRT were calculated as LRT = $-2(\ln\left( \frac{{Likelihood}_{reduced}}{{Likelihood}_{full}} \right)\sim\chi_{df}^{2} {df}_{full-reduced}$ (Gilmour et al. 2009; Supplemental Table 1).

The majority of traits showed evidence for interactions between GCA and the environment (Supplemental Table 1). Direct tests of SCA using the LRT (Supplemental Table 1) and the Wald test (Supplemental Table 2) were not significant. As there was a significant interaction between GCA and environment, but no evidence of SCA, the ambient and elevated O_3_ treatments were modeled separately to estimate heritability (methods described in main text). However, we also calculated heritability estimates for each trait across both O_3_ treatments (Supplemental Table 3). The standard errors of the heritability estimates were obtained with the delta method for both Models 2 and 3.

**References**

Schwarz, G. (1978) Estimating the dimension of a model. *Annals of Statistics*, 6(2), 461-464.

Coffman, C. J., Doerge, R.W., Simonsen, K.L., Nichols, K.M., Duarte, C.K., Wolfinger, R.D., McIntyre, L.M. (2005)*.* Model selection in binary trait locus mapping. *Genetics*, 170(3), 1281- 1297.

Gilmour, A.R., Gogel, B.J., Cullis, B.R., Thompson, R. (2009). ASReml user. Guide release 3.0. 372 p. VSN International Ltd., Hemel Hempstead, UK.

Searle SR (1971) Linear Models Wiley and Sons ISBN 047176950 (Chapter 4 Section 3)

**Supplemental Figures**

**Supplemental Figure 1.** Maximum and minimum air temperature (°C) and total precipitation (mm) in the 2016 and 2017 growing seasons. Grey bars show dates of gas exchange and reflectance measurements. Triangles represent days when irrigation was applied.


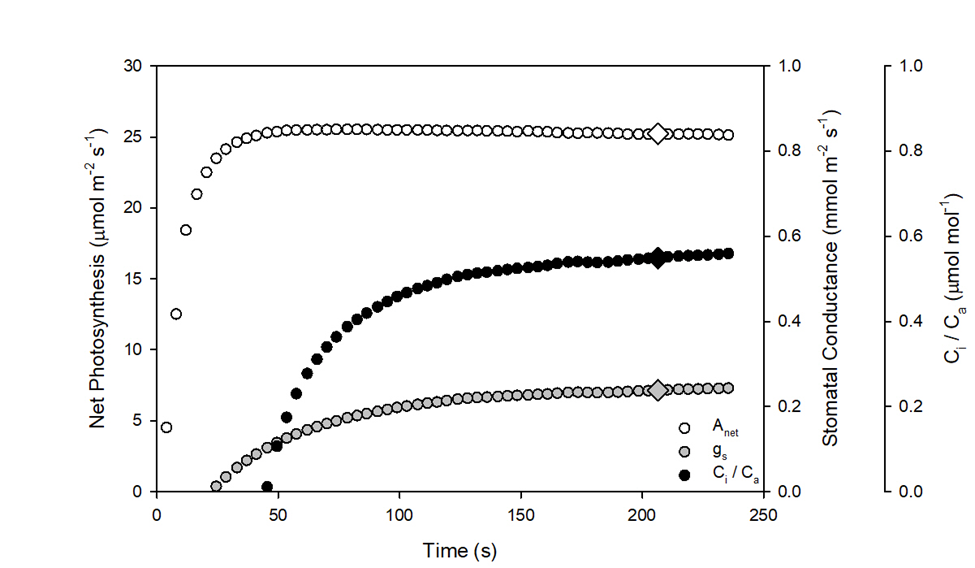


**Supplemental Figure 2.** Representative gas exchange data collected from hybrid Oh43 x MS71 grown at elevated [O_3_] in 2016. Net carbon assimilation (white circles), stomatal conductance (grey circles) and c_i_:c_a_ (black circles) were measured for 4 minutes and data were logged every 4 seconds. The diamond represents the mean value for the final minute of measurement, which was used for statistical analyses.

 **Supplemental Figure 3**. Measurements of stomatal conductance (g_s_) measured in maize hybrids. Measurements were made during the summer of 2016 (a) and 2017 (b) on plants grown in ambient O_3_ (white) and elevated O_3_ (black). Data are ordered based on g_s_ measured at ambient O_3_. Error bars represent 1 standard deviation (n=4).

**
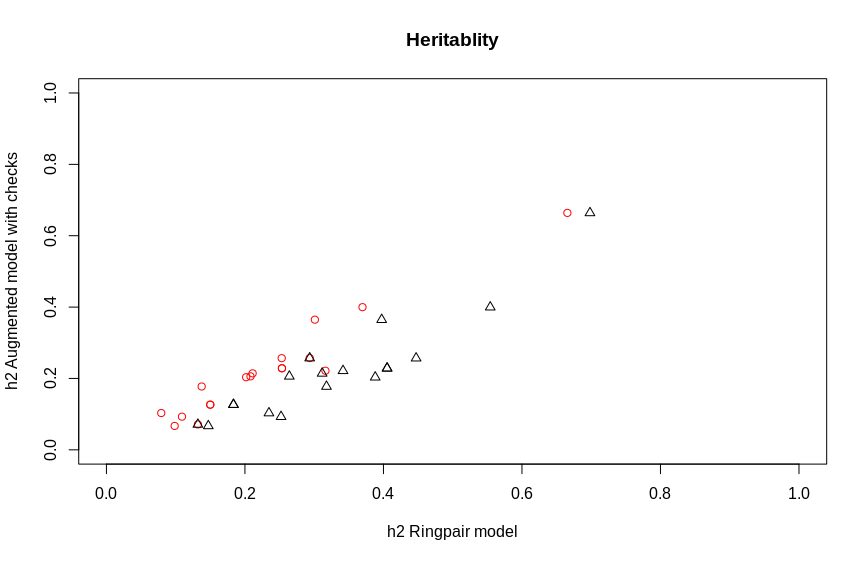
**

**Supplemental Figure 4.** Comparison of heritability the estimates of narrow (red circles) and broad (black triangles) sense heritability of different photosynthetic traits from the augmented design model originally used (checks as covariates) (y axis), and the model using the ring pair as a random effect (x axis).

**Supplemental Table 1.** Bayesian information criterion (BIC) values used in the evaluation of model fit in 2016 and 2017. Model numbers are described in the supplemental text above. In all comparisons the likelihood ratio tests (LRT) was carried out between full and reduced models and *p* values for the LRT ~ $\chi_{df}^{2} ; {df}_{full-reduced}$. Model 3, which includes an interaction between additive genetic variance and the environment, but no dominance effect, was the best fit model for the majority of the traits.

|  | **2016** | | | | | | | **2017** | | | | | | |
| --- | --- | --- | --- | --- | --- | --- | --- | --- | --- | --- | --- | --- | --- | --- |
|  | **Model fit- BIC** | | | **Models 1-2** | | **Models 2-3** | | **Model fit- BIC** | | | **Models 1-2** | | **Models 2-3** | |
|  | **Model 1** | **Model 2** | **Model 3** | **LRT** | **p-val** | **LRT** | **p-val** | **Model 1** | **Model 2** | **Model 3** | **LRT** | **p-val** | **LRT** | **p-val** |
| *A* | 3103.3 | 3103.3 | 3092.6 | 0.0 | 1.000 | 11.3 | 0.001 | 2055.9 | 2055.6 | 2019.1 | 0.4 | 0.527 | 37.2 | <0.0001 |
| g_s_ | -499.7 | -499.4 | -500.0 | 1.0 | 0.317 | 1.2 | 0.273 | -661.9 | -659.2 | -667.1 | 3.4 | 0.065 | 8.6 | 0.003 |
| c_i_:c_a_ | -700.6 | -701.2 | -710.8 | 0.1 | 0.752 | 10.3 | 0.001 | -761.6 | -760.1 | -769.2 | 2.1 | 0.147 | 9.8 | 0.002 |
| iWUE | 2105.6 | 2105.6 | 2078.4 | 0.0 | 1.000 | 27.9 | 0.000 | 3028.2 | 3028.9 | 3022.8 | 1.4 | 0.237 | 6.8 | 0.009 |
| V_maxm_ | 1414.7 | 1414 | 1395.2 | 0.0 | 1.000 | 19.5 | 0.000 | 1904.7 | 1906.2 | 1905.2 | 2.2 | 0.138 | 1.7 | 0.192 |
| C_hlm_ | 1112.2 | 1113.6 | 1093.5 | 2.1 | 0.147 | 20.8 | 0.000 | 1699.2 | 1702 | 1702.0 | 3.6 | 0.058 | 0.0 | 1.000 |

**Supplemental Table 2.** Table shows p-values from the test of dominance (SCA) effects for the null hypothesis that SCA is zero calculated using a Wald test from the model $\boldsymbol{y}_{\boldsymbol{ijkm}}\boldsymbol{=u+}\boldsymbol{T}_{\boldsymbol{i}}\boldsymbol{+}\boldsymbol{C}_{\boldsymbol{ijk}}\boldsymbol{+}\boldsymbol{G}_{\boldsymbol{m}}\boldsymbol{+}{\boldsymbol{T}_{\boldsymbol{i}}\boldsymbol{*G}}_{\boldsymbol{m}}\boldsymbol{+}\boldsymbol{\epsilon}_{\boldsymbol{ijkm}}$. * variance component estimate was 0

| **Trait** | **2016** | **2017** |
| --- | --- | --- |
| *A* | 0.477 | 0.278 |
| g_s_ | 0.193 | 0.073 |
| c_i_:c_a_ | 0.370 | 0.115 |
| iWUE | * | 0.158 |
| V_maxm_ | 0.487 | 0.110 |
| Chl_m_ | 0.115 | 0.070 |
